# Cytosolic nucleic acid sensing triggers type I interferon activation *via* the Hippo kinase LATS1

**DOI:** 10.1016/j.jbc.2026.111204

**Published:** 2026-01-23

**Authors:** Tim Nass, Anna D. Reichardt, Saba R. Aliyari, Emily Tom, Gage LeMunyan, Michelle S. Parvatiyar, Genhong Cheng, Kislay Parvatiyar

**Affiliations:** 1 Department of Microbiology & Immunology, Tulane University School of Medicine, New Orleans, Louisiana, USA; 2 Department of Microbiology, Immunology, and Molecular Genetics, University of California, Los Angeles, California, USA; 3 Medical Scientist Training Program, David Geffen School of Medicine, UCLA, Los Angeles, California, USA; 4 Department of Health, Nutrition, and Food Sciences, Florida State University, Tallahassee, Florida, USA; 5 Jonsson Comprehensive Cancer Center, UCLA, Los Angeles, California, USA

**Keywords:** large tumor suppressor 1 (LATS1), interferon regulatory factor 3 (IRF3), TANK binding kinase 1 (TBK1), type I interferon (IFN-I), Hippo signaling, innate antiviral signaling, pattern recognition receptors (PRRs)

## Abstract

Innate immune detection of viral genomes *via* nucleic acid-sensing pattern recognition receptors in the cytosolic compartment triggers the production of type I interferons (IFN-I) to coordinate a cellular antiviral state to limit viral replication and spread. While IFN-I induction is controlled primarily by the IRF3 transcription factor, which undergoes phosphorylation-dependent activation *via* the virus-activated kinase, TBK1, the mechanisms underlying how TBK1 signaling is achieved remain incompletely understood. Here, we report that viral infection or cytosolic delivery of nucleic acids elicits the activation of a primordial Hippo signaling pathway that is known to control organ size and tissue homeostasis. We identify the Hippo core component, LATS1 kinase to necessitate TBK1 dependent signaling as cells treated with a pharmacological inhibitor of LATS1 or cells from *Lats1*^*−/−*^ mice displayed impaired TBK1-IRF3 signal activities and defective IFN-I induction upon cytosolic nucleic acid stimulation. Consequently, LATS1-deficient cells harbored elevated viral titers in comparison to WT control cells. Mechanistically, LATS1 associated with TBK1 upon cytosolic nucleic acid stimulation and promoted TBK1 signaling and activation in a kinase-dependent manner. Altogether, our findings reveal that cytosolic nucleic acid-sensing pathways elicit Hippo/LATS1 activation to govern TBK1 signaling events to result in IFN-I activation.

The innate immune system operates as the initial line of defense against microbial infections including those caused by viral pathogens. Upon infection, innate immune cells elicit the production of type I interferon (IFN-I) cytokines (*e.g.* IFNα/β) which coordinate an antiviral gene program to interfere with viral replication and spread (1, 2, 3). The expression of IFN-I is tightly controlled, primarily by the interferon regulatory factor 3 (IRF3) transcription factor which is activated upon phosphorylation by the upstream kinase, TANK-binding kinase 1 (TBK1) (2, 4, 5). Signaling to TBK1-IRF3 is largely governed by germ-line encoded pattern recognition receptors (PRRs) that sense viral genomes or nucleic acids in the cytosolic compartment (4, 5, 6). RNA virus genomes are detected by retinoic acid inducible gene I (RIG-I) or melanoma differentiation associated gene 5 (MDA5) PRRs which signal to TBK1-IRF3 *via* the adaptor protein, mitochondrial antiviral signaling (MAVS) in what is known as the cytosolic RNA sensing pathway. In contrast, DNA virus genomes are sensed by cyclic guanosine adenosine monophosphate synthase (cGAS), DEAD-box helicase 41 (DDX41), or interferon gamma-inducible protein 16 (IFI16) PRRs which instead utilize the stimulator of interferon genes (STING) adaptor to activate TBK1-IRF3 in a cytosolic DNA sensing pathway (4, 5, 7, 8, 9).

A distinct, evolutionarily conserved Hippo signaling module functions as a molecular network to control organ size and tissue homeostasis by regulating cellular proliferation and apoptosis. It also plays a crucial role in stem cell renewal and tissue regeneration, ensuring proper development and maintenance of cellular health (10, 11, 12). Under basal conditions (Hippo off), co-transcriptional regulators Yes-associated protein (YAP) and transcription co-activator with PDZ-binding motif (TAZ) reside in a nuclear complex with transcriptional enhanced associated domain 1 to 4 (TEAD1-4) transcription factors to drive the expression of pro-proliferative and pro-survival genes. In response to various extracellular and/or intracellular cues such as cell-cell contact, mechanical stress, and changes in the cellular microenvironment (Hippo on), an upstream core kinase complex containing mammalian STE-20 like 1/2 (MST1/2) and large tumor suppressor 1/2 (LATS1/2) undergo phosphorylation dependent activation to result in downstream YAP/TAZ phosphorylation and consequent inhibition *via* cytoplasmic sequestration and proteasomal degradation, ultimately preventing uncontrolled TEAD1-4 dependent gene expression (13, 14, 15, 16, 17).

Dysregulation of the Hippo pathway has been linked to various disorders, including liver disease and cancer. In liver disease, for example, disruptions in Hippo signaling contribute to fibrosis and impaired tissue regeneration. Additionally, in cancer, aberrant Hippo signaling leads to unchecked activation of YAP/TAZ, promoting tumor growth and metastasis (10, 12, 15). Emerging studies have further highlighted the role of the Hippo pathway in innate immune regulation during viral infections, in which a key component of the Hippo pathway was shown to modulate cytosolic nucleic acid-sensing PRR signaling and the IFN-I response. Indeed, the YAP co-transcriptional activator was demonstrated to also function as a steady-state negative regulator of IFN-I induction by restricting TBK1/IRF3 signaling events, and in a Hippo effector (*e.g.* TEAD1-4) independent manner (18, 19). Upon viral infection or cytosolic nucleic acid stimulation, YAP blockade of IFN-I was additionally shown to be lifted by way of phospho-inhibition of YAP *via* the TBK1 homolog, inhibitory kappa B kinase epsilon (IKKε), to result in YAP degradation (19). While a role for an IKKε-YAP axis to modulate activation of IFN-I has been demonstrated, it remains unclear whether canonical Hippo pathway activation is elicited during innate immune signaling to facilitate antiviral host defenses.

Herein, we demonstrate that viral infection or cytosolic nucleic acid sensing coincides with Hippo pathway signaling and YAP inactivation *via* its canonical kinase, LATS1. Mechanistically, cytosolic PRR signaling triggers LATS1 phosphorylation to result in subsequent YAP degradation. Furthermore, we show that cytosolic PRR engagement causes LATS1 to associate with TBK1 and license TBK1 activation in a kinase-dependent manner. This ultimately promotes signaling to IRF3 resulting in IFN-I activation to confer innate antiviral host defenses. Altogether, our findings identify an essential role for classical Hippo pathway signaling *via* LATS1 in facilitating innate antiviral immune signaling.

## Results

### Virus infection and cytosolic nucleic acid sensing trigger Hippo pathway activation in a LATS1-dependent manner

**Figure 1:**
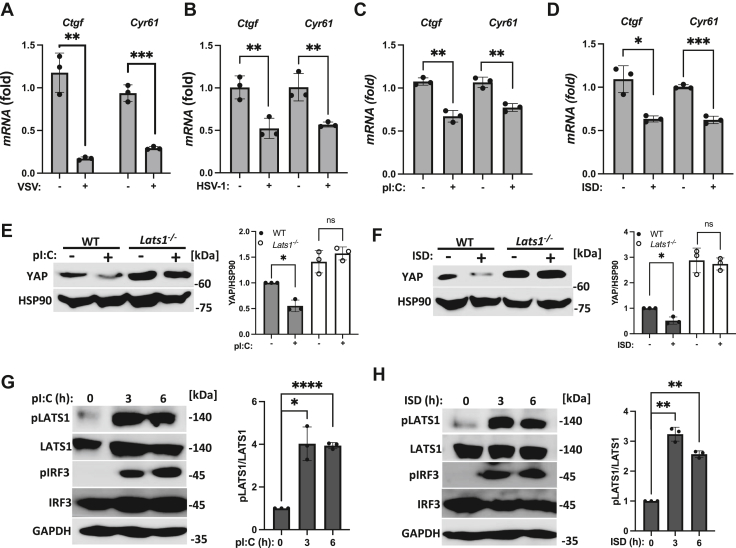
**Virus infection and cytosolic nucleic acid sensing trigger Hippo pathway activation in a LATS1-dependent manner.***A* and *B*, quantitative real-time PCR (qPCR) analysis of TEAD regulated genes Ctgf and Cyr61 in murine embryonic fibroblast (MEF) cells after infection with (*A*) vesicular stomatitis virus (VSV) (MOI 0.75; 24 h) or (*B*) herpes simplex virus-1 (HSV-1) (MOI 0.5; 8 h). *C* and *D*, qPCR of Ctgf and Cyr61 expression in MEF cells transfected with pI:C (*C*) or ISD (*D*) (2 μg/ml each; 3 h). *E* and *F*, immunoblot analysis (*left panels*) and relative quantification (*right panels*) of YAP expression in WT or *Lats1*^*−/−*^ MEF cells transfected with pI:C (*E*) or ISD (*F*) (2 μg/ml each; 8 h). *G* and *H*, immunoblot analysis (*left panels*) and relative quantification (*right panels*) of LATS1 (T1079) and IRF3 (S396) phosphorylation in MEF cells transfected with pI:C (*G*) or ISD (*H*) (2 μg/ml each) for the indicated times. Statistical significance was determined using Student’s *t* test (∗∗∗*p* < 0.001, ∗∗*p* < 0.01, and ∗*p* < 0.05).

The Hippo pathway co-transcriptional activator, YAP was previously shown to function as a negative regulator of IFN-I, as the promotion of YAP degradation *via* Hippo pathway-induced serum starvation or increased cellular density prior to viral infection boosted IFN-I activation when compared to virally infected cells that were not starved or overcrowded. To obtain a better understanding of the functional relevance of the Hippo pathway in the context of innate antiviral signaling, we first explored whether virus infection alone could trigger Hippo pathway activation. Utilizing immortalized murine embryonic fibroblasts (MEFs) as these cells harbor intact cytosolic nucleic acid sensing machinery and Hippo pathway signaling components, we found that cells infected with the RNA virus, vesicular stomatitis virus (VSV) (Fig. S1*A*), or the DNA virus, herpes simplex virus type 1 (HSV-1) (Fig. S1*B*), exhibited an increase in YAP phosphorylation, resulting in the reduction of YAP protein expression (Fig. S1, *C* and *D*) and the suppression of the YAP-TEAD1-4 regulated genes, connective tissue growth factor (CTGF) and cysteine-rich angiogenic inducer 61 (CYR61) (Fig. 1, *A* and *B*), signifying induction of the Hippo pathway. Like RNA or DNA virus infections, cytosolic delivery of the RNA sensing pathway inducing ligand, polyinosinic-polycytidylic acid (poly(I:C)) (Fig. S1*E*), or the DNA pathway inducing ligand, immunostimulatory DNA (ISD) (Fig. S1*F*), resulted in signaling to YAP to consequently inhibit CTGF and CYR61 expression (Fig. 1, *C* and *D*). In addition to cytosolic RNA and DNA sensing pathways, the membrane bound toll-like receptor 4 (TLR4) can also activate IFN-I upon receptor ligation (20, 21). Consistently, cells treated with the TLR4 agonist, lipopolysaccharide (LPS), also displayed Hippo pathway activation (Fig. S1*G*). Given that in canonical Hippo signaling, LATS1 functions as the prominent kinase that phosphorylates YAP to instigate its degradation, we next sought to determine whether LATS1 plays a role in conferring YAP degradation in the cytosolic RNA and DNA sensing pathways. Indeed, LATS1-deficient MEFs (Fig. S1*H*) that were virally infected (Fig. S1*I*) or transfected with poly(I:C) or ISD (Fig. 1, *E* and *F*) were deficient in promoting YAP degradation as their levels of YAP remained largely intact compared to WT MEFs. These observations indicate that YAP inhibition/degradation events are dependent on LATS1 in the RNA and DNA sensing pathways which prompted us to explore if cytosolic nucleic acid sensing could also trigger LATS1 activation. Similar to conventional Hippo pathway signaling, cytosolic delivery of poly(I:C) or ISD promoted the phospho-activation of LATS1 (Fig. 1, *G* and *H*), suggesting that the RNA and DNA sensing pathways utilize LATS1 to antagonize YAP function. Taken together, our findings reveal that cytosolic nucleic acid sensing elicits Hippo pathway activation in a LATS1-dependent manner.

### The pharmacological inhibition of LATS impedes cytosolic nucleic acid-sensing-mediated activation of IFN-I

**Figure 2:**
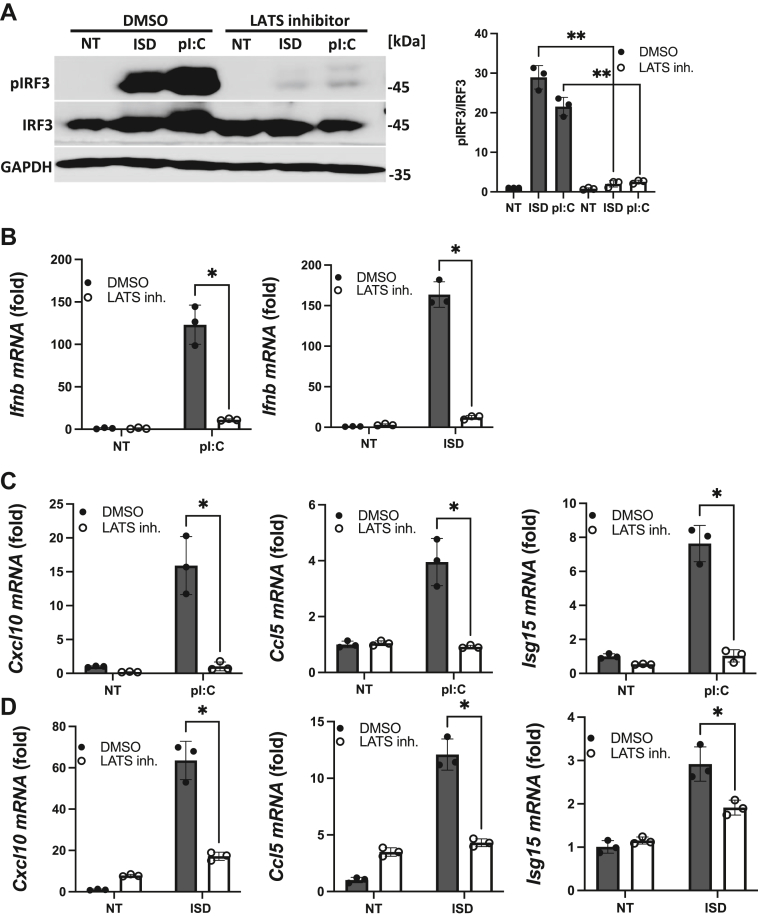
**Pharmacological Inhibition of LATS impedes cytosolic nucleic acid sensing PRR activation of IFN-I.***A*, immunoblot analysis (*left panel*) and relative quantification (*right panel*) of IRF3 (S396) phosphorylation in MEF cells pretreated with vehicle control (DMSO) or LATS inhibitor (20 μM; 2.5 h) then transfected with pI:C or ISD (2 μg/ml each; 3 h). *B*, qPCR analysis of Ifnb mRNA in MEF cells pretreated with DMSO or LATS inhibitor as in *A*, followed by pI:C transfection (2 μg/ml; 3.5 h) (*left panel*) or ISD transfection (2 μg/ml; 4 h) (*right panel*). *C* and *D*, qPCR of Cxcl10, Ccl5, and Isg15 mRNA in MEF cells pretreated with DMSO or LATS1 inhibitor followed by pI:C transfection as in *B*, *left panel* (*C*) or ISD as in *B*, *right panel* (*D*). Statistical significance was determined using student’s *t* test (∗∗∗*p* < 0.001, ∗∗*p* < 0.01, and ∗*p* < 0.05).

Given that cytosolic nucleic acid sensing coincides with LATS1 activation, we wanted to address the possibility of a functional role for LATS1 in controlling the IFN-I response. We utilized LATS-IN-1, a potent and ATP-competitive inhibitor of LATS1/2 kinases that blocks Hippo pathway activation (22) (Fig. S2*A*). MEF cells transfected with ISD or poly(I:C) displayed robust phospho-activation of the IRF3 transcription factor, whereas MEFs pretreated with the LATS inhibitor were highly impaired in promoting IRF3 activation (Fig. 2*A*). Consequently, cells pretreated with the LATS inhibitor were significantly defective in inducing IFN-I (*e.g.* IFN-β) compared to vehicle control treated cells (Fig. 2*B*). In addition to IRF3-mediated induction of IFN-I, cytosolic nucleic acid-sensing pathways can trigger NF-κB-dependent activation of pro-inflammatory cytokines, including TNFα (23, 24). Notably, pharmacological inhibition of LATS diminished the expression of TNFα upon stimulation of the RNA- and DNA-sensing pathways (Fig. S2*B*). IFN-I cytokines engage the IFNα/β receptor on neighboring cells to elicit the activation of interferon-stimulated genes (ISGs) which play a cardinal role in sterilizing host cells during viral infections (6, 25, 26). As we found that LATS inhibition suppressed IFN-I induction upon cytosolic nucleic acid sensing, the presence of LATS inhibitor accordingly blocked the activation of ISGs including C-X-C motif chemokine ligand 10 (CXCL10), C-C motif chemokine ligand 5 (CCL5) and ISG15 after RNA pathway (Fig. 2*C*) or DNA pathway (Fig. 2*D*) stimulation. In addition to murine cells, we extended our studies to human U937 monocytes to also find that these cells treated with LATS inhibitor were impaired in promoting IRF3 phospho-activation (Fig. S2*C*) to subsequently result in defective IFN-I and ISG responses in the RNA pathway (Fig. S2*D*). Likewise, activation of the DNA pathway in human monocytes was significantly blunted in the presence of LATS inhibitor, resulting in defective STING signaling (Fig. S2*E*) and dampened IFN-I and ISG inductions (Fig. S2*F*). These results point to an essential role for LATS in facilitating the IFN-I response in the cytosolic RNA and DNA sensing pathways.

### LATS1 is required for IFN-I activation

**Figure 3:**
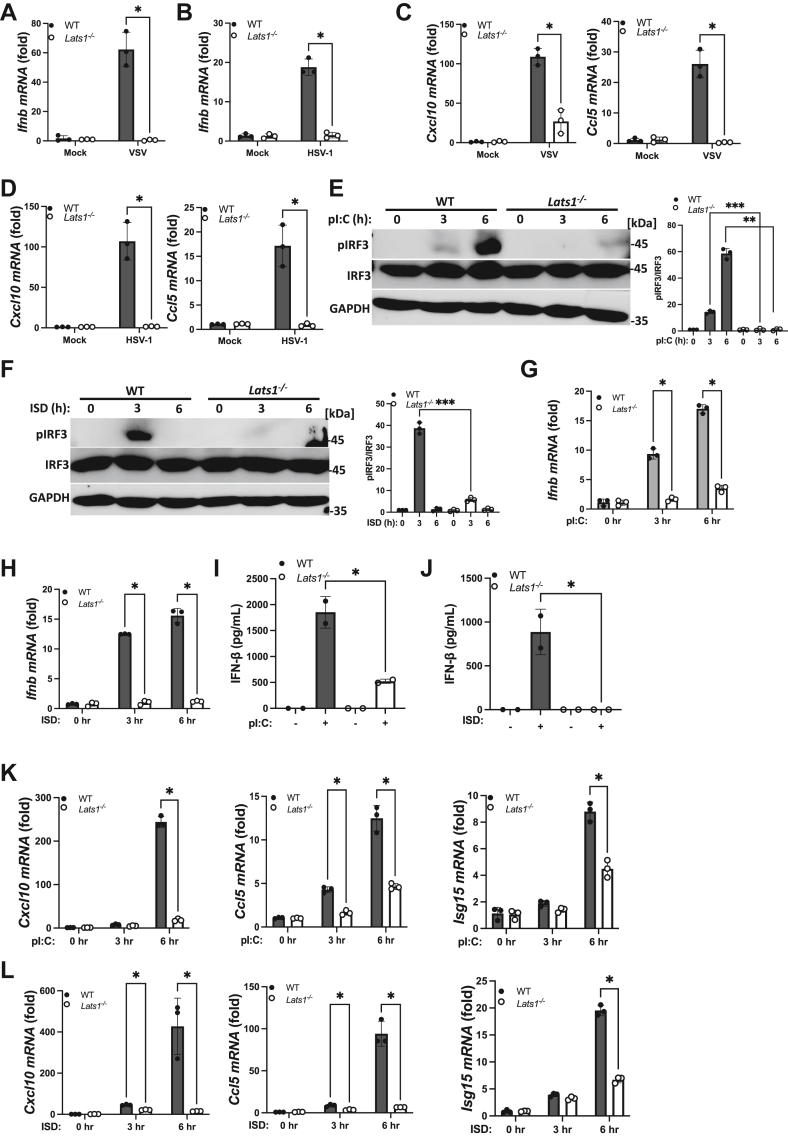
**LATS1 is required for IFN-I activation.***A* and *B*, qPCR analysis of Ifnb mRNA expression in WT and *Lats1*^*−/−*^ MEF cells infected with VSV (MOI 0.5) (*A*) or HSV-1 (MOI 0.1) for 24 h. *C* and *D*, qPCR analysis of Cxcl10 and Ccl5 mRNA in WT and *Lats1*^*−/−*^ MEF cells infected with VSV as in panel *A* (*C*) or HSV-1 as in panel *B* (*D*). *E* and *F*, immunoblot analysis (*left panels*) and relative quantification (*right panels*) of IRF3 (S396) phosphorylation in WT and *Lats1*^*−/−*^ MEF cells transfected with pI:C (*E*) or ISD (*F*) (2 μg/ml each) for the indicated times. *G* and *H*, qPCR analysis of Ifnb mRNA expression in WT and *Lats1*^*−/−*^ MEF cells transfected with pI:C (*G*) or ISD (*H*) (2 μg/ml each) for the indicated times. *I* and *J*, ELISA for IFN-β secretion in WT and *Lats1*^*−/−*^ MEF cells transfected with pI:C (*I*) or ISD (*J*) (2 μg/ml each; 6 h). *K* and *L*, qPCR analysis of Cxcl10, Ccl5, and Isg15 mRNA in WT and *Lats1*^*−/−*^ MEF cells transfected with pI:C (*K*) or ISD (*L*) (2 μg/ml each) for the indicated times. Statistical significance was determined using Student’s *t* test (∗∗∗*p* < 0.001, ∗∗*p* < 0.01, and ∗*p* < 0.05).

Our data utilizing a pharmacological inhibitor of LATS suggest that LATS kinases function as positive regulators of IFN-I downstream of cytosolic nucleic acid sensing pattern recognition receptors. To further validate a role of LATS in a genetic model, we investigated the contribution of LATS1 to innate immune signaling in the RNA- and DNA-sensing pathways utilizing MEF cells obtained from *Lats1*^*−/−*^ mice. While infections with the RNA viruses, VSV (Fig. 3*A*) and Sendai virus (SeV) (Fig. S3*A*), or the DNA virus, HSV-1 (Fig. 3*B*) caused a robust induction of IFN-I in WT MEFs, *Lats1*^*−/−*^ MEFs displayed significantly lower IFN-I levels upon viral infections. Consequently, LATS1 deficient MEFs showcased lower levels of ISGs upon viral infections compared to WT MEF cells (Figs. 3, *C* and *D* and S3*B*). As IFN-I activation is primarily controlled by the IRF3 transcription factor, we next sought to determine if LATS1 plays a role in facilitating signaling to IRF3 in the RNA and DNA sensing pathways. WT MEFs transfected with poly(I:C) or ISD potently triggered the phospho-activation of IRF3, whereas *Lats1*^*−/−*^ MEFs were largely defective in promoting IRF3 activation during cytosolic nucleic acid stimulations (Fig. 3, *E* and *F*). In line with these observations, IFN-I induction (Figs. 3, *G* and *H* and S3*C*) and subsequent IFN-I cytokine production (Fig. 3, *I* and *J*) were significantly blunted in LATS1-deficient MEFs in comparison to WT cells, resulting in impaired activation of downstream ISGs (Figs. 3, *K* and *L* and S3). Furthermore, RNA and DNA pathway-mediated activation of NF-κB (Fig. S3, *E* and *F*) and the subsequent expression of pro-inflammatory cytokines (Fig. S3*G*) were also curbed in *Lats1*^*−/−*^ MEFs. To determine if LATS1 plays a role in controlling the IFN-I response in human cells, we utilized CRISPR-Cas9 technology to delete LATS1 in human A549 lung epithelial cells (Fig. S3*H*). While viral infection or poly(I:C) transfection elicited the activation of IFN-I in control A549 cells, LATS1 KO A549 cells exhibited significantly reduced IFN-I, pro-inflammatory cytokine, and ISG expressions (Fig. S3*I*). LATS1 and its homolog, LATS2, share extensive sequence similarity within their kinase domains (∼85% similarity) and can converge on some similar cellular processes. Indeed, we found that the expression of LATS2 was intact in *Lats1*^*−/−*^ MEF cells (Fig. S3*J*), and that the treatment of these cells with the LATS inhibitor (that targets the kinase activity of both LATS1 and LATS2) resulted in reduced IFN-I activation (Fig. S3*K*), suggesting that LATS2 may play a non-redundant role with LATS1 in facilitating the IFN-I response in cytosolic nucleic acid sensing pathways. Taken together, these results align with our pharmacological data to reveal an essential role for LATS1 in facilitating the IFN-I response in the cytosolic RNA and DNA sensing pathways.

### Cellular antiviral host defenses are dependent on LATS1

**Figure 4:**
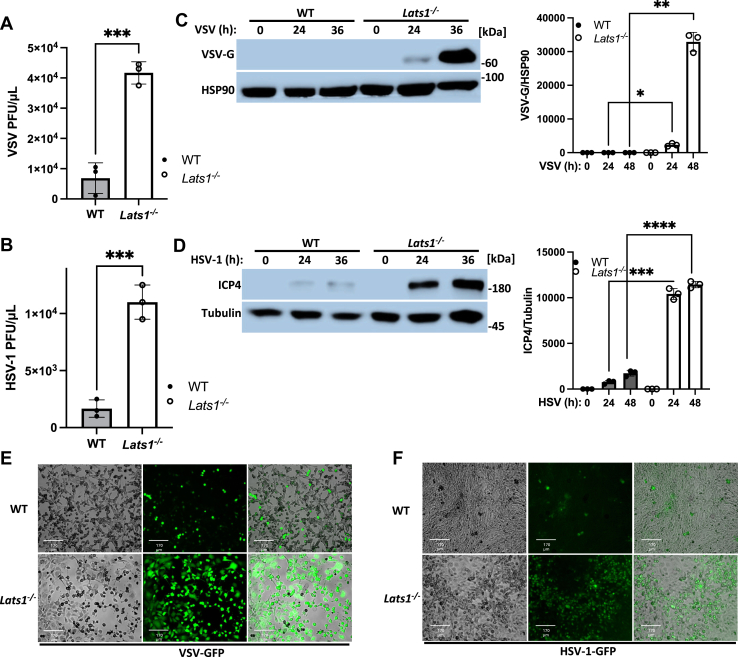
**Cellular antiviral host defenses are dependent on LATS1.***A* and *B*, plaque assay for viral titers from supernatants of WT and *Lats1*^*−/−*^ MEF cells infected with VSV (MOI 0.1) (*A*) or HSV-1 (MOI 0.4) for 16 h. *C*, immunoblot analysis (*left panel*) and relative quantification (*right panel*) for VSV encoded glycoprotein, G (VSV-G) in WT and *Lats1*^*−/−*^ MEF cells infected with VSV (MOI 0.1) for the indicated times. *D*, immunoblot analysis (*left panel*) and relative quantification (*right panel*) of HSV-1 encoded immediate early transcription factor, ICP4 (HSV-1 ICP4) expression in WT and *Lats1*^*−/−*^ MEF cells infected with HSV-1 (MOI 0.1) for the indicated times. *E* and *F*, fluorescent imaging of WT and *Lats1*^*−/−*^ MEF cells infected with VSV-GFP (MOI 0.1) (*E*) or HSV-1-GFP (MOI 0.1) (*F*) for 24 h. *Left* to *right*: bright field, GFP, merged channels. Scale bar = 170 μm. Statistical significance was determined using student’s *t* test (∗∗∗*p* < 0.001, ∗∗*p* < 0.01, and ∗*p* < 0.05).

Given our identification that LATS1 operates as a positive regulator of the IFN-I response in the RNA- and DNA-sensing pathways, and that IFN-I plays a critical role in restricting viral replication and spread, we next sought to determine the impact of LATS1 in controlling viral infection. Upon RNA or DNA virus infection, *Lats1*^*−/−*^ MEFs exhibited elevated viral titers compared to WT MEFs as determined by standard plaque assay (Fig. 4, *A* and *B*). Consistent with these observations, MEF cells lacking LATS1 showcased increased expression of the VSV encoded glycoprotein G (Fig. 4*C*) and the HSV-1 encoded protein, ICP4 (Fig. 4*D*), which corresponded with increased susceptibility to viral infection when compared to control infected cells (Fig. 4, *E* and *F*). Collectively, these results indicate that LATS1 plays a crucial role in cellular antiviral host defenses by controlling both RNA and DNA virus replication.

### LATS1 operates at the level of TBK1

**Figure 5:**
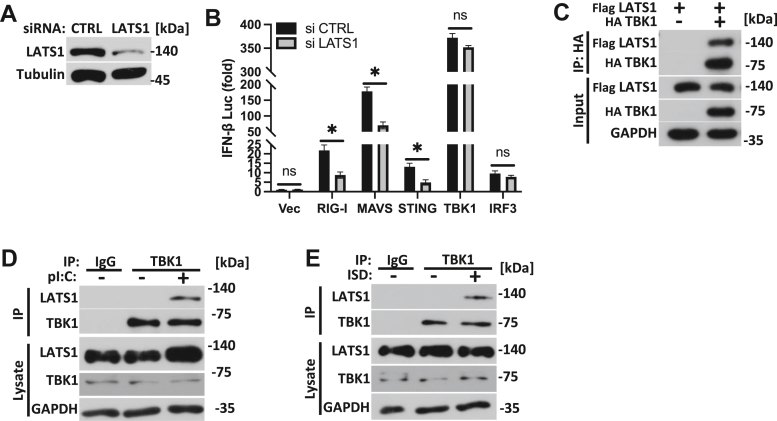
**LATS1 operates at the level of TBK1.***A*, immunoblot analysis of LATS1 expression in HEK 293T cells treated with control siRNA or siRNAs targeting LATS1 for 24 h. *B*, IFN-β luciferase reporter assay in HEK 293T cells treated with control siRNA or siRNAs targeting LATS1 as in *A*, followed by transfection of plasmids encoding RIG-I, MAVS, STING, TBK1, and IRF3. *C*, co-immunoprecipitation and immunoblot of FLAG-LATS1 and HA-TBK1 co-transfected in HEK 293T cells. *D* and *E*, immunoblot analysis of TBK1-LATS1 interactions in MEF cells transfected with pI:C (*D*) or ISD (*E*) (2.5 μg/ml each; 2 h). Statistical significance was determined using student’s *t* test (∗∗∗*p* < 0.001, ∗∗*p* < 0.01, and ∗*p* < 0.05).

As our data found a key role for LATS1 in facilitating IFN-I activation in the RNA and DNA sensing pathways, we next wanted to determine if LATS1 could engage RNA and/or DNA sensing PRR signaling components. Utilizing small-interfering RNA (siRNA) targeting LATS1 in HEK 293T cells (Fig. 5*A*), we found that while LATS1 was necessary to promote RIG-I, MAVS, and STING-dependent activation of IFN-I, downstream TBK1 (and IRF3) mediated activation of IFN-I was not significantly impaired in LATS1-silenced cells (Fig. 5*B*). Similarly, *Lats1*^*−/−*^ MEFs were defective in facilitating MAVS and STING, but not TBK1-mediated activation of IFN-I in comparison to WT control MEFs (Fig. S4*A*) altogether suggesting LATS1 to operate at the level of TBK1, a shared signaling component in the RNA and DNA sensing pathways. Ectopic expression of LATS1 with TBK1 further showed that LATS1 could interact with TBK1 (Fig. 5*C*). Under physiological conditions, LATS1 is associated with TBK1 upon cytosolic nucleic acid stimulation, suggesting their endogenous interactions occur upon RNA or DNA pathway engagement (Fig. 5, *D* and *E*).

### LATS1 facilitates TBK1 activation and signaling events

**Figure 6:**
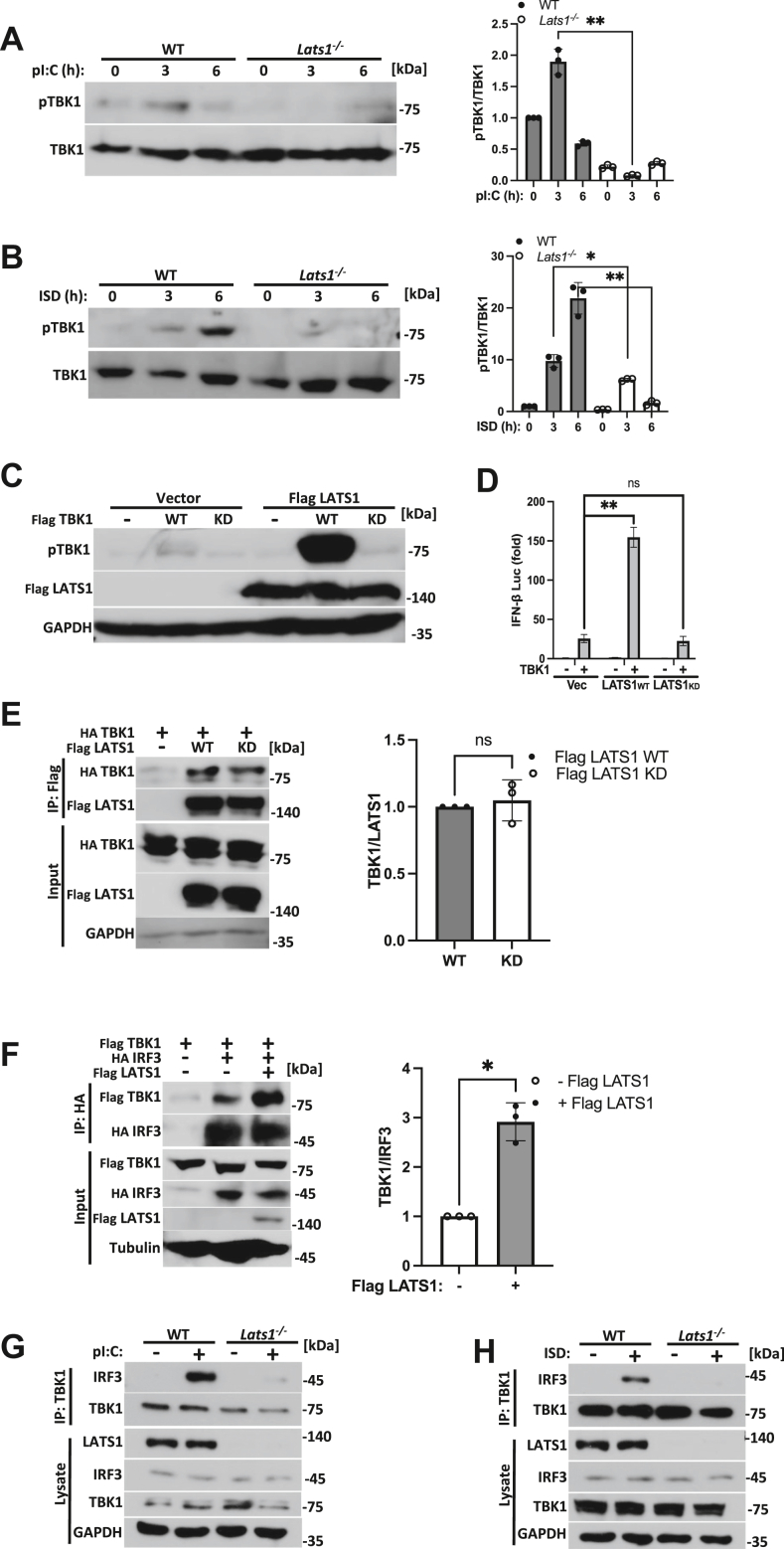
**LATS1 facilitates TBK1 activation and signaling events.***A* and *B*, immunoblot analysis (*left panels*) and relative quantification (*right panels*) of TBK1 (S172) phosphorylation in WT and *Lats1*^*−/−*^ MEF cells transfected with pI:C (*A*) or ISD (*B*) (2 μg/ml each) for the indicated times. *C*, immunoblot analysis of TBK1 (S172) phosphorylation in HEK 293T cells co-transfected with plasmids encoding WT TBK1 or kinase dead (KD) TBK1 (K38A) in the absence or presence of LATS1. *D*, IFN-β luciferase reporter assay in HEK 293T cells co-transfected with plasmids encoding TBK1 in the presence of WT LATS1 or kinase dead (KD) LATS1 (D846A). *E*, co-immunoprecipitation and immunoblot (*left panel*) and relative quantification (*right panel*) of WT LATS1-TBK1 and KD LATS1-TBK1 interactions in HEK 293T cells co-transfected with the indicated plasmids. *F*, co-immunoprecipitation and immunoblot (*left panel*) and relative quantification (*right panel*) of TBK1-IRF3 interactions in the absence or presence of LATS1 in HEK 293T cells co-transfected with the indicated plasmids. *G* and *H*, immunoblot analysis of TBK1-IRF3 interactions in WT and *Lats1*^*−/−*^ MEF cells transfected with pI:C (*G*) or ISD (*H*) (2 μg/ml each; 2.5 h). Statistical significance was determined using student’s *t* test (∗∗∗*p* < 0.001, ∗∗*p* < 0.01, and ∗*p* < 0.05).

As we found that LATS1 interacts with TBK1, we next extended our studies to address whether LATS1 was important for governing TBK1 activation in the RNA and DNA sensing pathways. Cytosolic delivery of poly(I:C) or ISD elicited TBK1 phospho-activation in WT cells, whereas *Lats1*^*−/−*^ MEFs were notably impaired in signaling to TBK1 upon RNA or DNA pathway stimulation (Fig. 6, *A* and *B*). Given that LATS1 possesses kinase activity (which is known to facilitate Hippo signaling) and that TBK1 phospho-activation was markedly absent in LATS1-deficient cells, we sought to determine whether LATS1 could operate as a kinase to phosphorylate TBK1. To this end, we co-transfected HEK 293T cells with plasmids encoding TBK1 in the absence or presence of LATS1 followed by immunodetection for phospho-TBK1. As TBK1 over-expression can promote auto-phosphorylation and potentially obfuscate the impact of LATS1 kinase activity, we additionally expressed kinase dead (K38A) TBK1 (TBK1 KD) in the presence of LATS1. While the expression of LATS1 amplified WT TBK1 phosphorylation, LATS1 could not drive the phosphorylation of TBK1 KD, suggesting that LATS1 likely promotes TBK1 auto-phosphorylation but does not function as a kinase for TBK1 (Fig. 6*C*). To determine whether LATS1 kinase activity is dispensable to facilitate TBK1 signaling, HEK 293T cells were co-transfected with TBK1 in the absence or presence of LATS1 or a kinase inactive (D846A) LATS1 plasmid construct (LATS1 kinase dead; LATS1 KD) (Fig. S4*B*). IFN-I reporter activity assays revealed that WT LATS1 synergized with TBK1 to enhance TBK1 mediated activation of IFN-I. However, kinase inactive LATS1 failed to synergize with TBK1 and could not enhance IFN-I activity (Fig. 6*D*). Notably, LATS1 KD interactions with TBK1 remained intact, suggesting that LATS1 did not require kinase activity to associate with TBK1 (Fig. 6*E*). To further understand how LATS1 confers TBK1 signaling to IFN-I, we examined if LATS1 played a role in fostering TBK1-IRF3 interactions. In gain of function experiments, LATS1 expression enhanced complex formations between TBK1 and IRF3 (Fig. 6*F*). Alternatively, RNA and DNA pathway triggered TBK1-IRF3 interactions were significantly defective in *Lats1*^*−/−*^ MEFs in comparison to WT MEFs (Fig. 6, *G* and *H*). Taken altogether, our data indicate that LATS1 promotes TBK1 activation and downstream signaling events in a kinase dependent manner to mediate IRF3 activation and IFN-I induction.

## Discussion

In this study, we provide evidence that viral infections and cytosolic nucleic acid sensing pathways, both of which elicit innate antiviral signaling to induce IFN-I, coincides with the activation of Hippo signaling. The Hippo co-transcriptional regulator, YAP (as well as its paralog, TAZ) was previously shown to function as a negative regulator of IFN-I activation downstream of cytosolic nucleic acid sensing pathways by targeting TBK1-IRF3 (18, 19). Additional studies revealed that upon viral infection, the TBK1 homolog, IKKε phosphorylated YAP at a non-conventional site (YAP S403) to result in the lysosomal degradation of YAP, consequently restricting its ability to antagonize TBK1-IRF3 (19). In contrast, another study found IKKε to phospho-inhibit LATS1/2 to suppress Hippo signaling (27). Our work, however, identifies a role for the classical Hippo kinase, LATS1 to undergo phospho-activation (LATS1 T1079) during cytosolic nucleic acid sensing to result in conventional YAP phospho-inhibition (YAP S127) and degradation in a LATS1-dependent manner. Given that the classical LATS1-YAP axis is known to confer YAP proteasomal degradation while the IKKε-YAP axis was shown to promote YAP lysosomal degradation, future studies will be necessary do decipher the specific degradation pathways that antagonize YAP during viral infection.

In the Hippo signaling pathway, LATS1 phospho-activation is typically mediated by the MST1/2 kinases (13, 14, 15). A previous study found that activation of the cytosolic DNA sensing pathway could cause IRF3 dependent transactivation of the *MST1* gene promoter to induce MST1 expression and upregulation to potentially facilitate downstream Hippo signal activation (28). Paradoxically, MST1 was reported to inhibit IFN-I activation by inactivating IRF3 in a kinase-dependent manner (29). Alternatively, mitogen-activated protein kinase kinase kinase kinase (MAP4K) family members as well as thousand-and-one amino acid protein kinases (TAOKs) have been shown to activate LATS1 and promote Hippo signaling (30, 31). As such, it may be possible that viral infection or cytosolic nucleic acid stimulation triggers LATS1 activation *via* these atypical kinases while the mechanisms by which MST1 modulates IFN-I activation will require further exploration.

Our data revealed an essential role for LATS1 in facilitating IFN-I activation upon viral infection and cytosolic nucleic acid sensing. As such, we expectedly found that LATS1-deficient cells were impaired in inducing IFN-I-stimulated genes (ISGs) which largely function as antiviral effectors to suppress viral replication. Consequently, *Lats1*^*−/−*^ MEF cells harbored elevated viral loads compared to WT cells. Consistent with our observations, a recent study described a critical role for LATS1 in conferring innate immunity to viral infections. As *Lats1*^*−/−*^ mice display dysfunctions in mammary gland development, infertility, and growth impairment (32), *Lats1*^*+/−*^ mice were utilized to examine the contribution of LATS1 to antiviral host defense to reveal that these animals succumbed faster to viral infection and presented with increased viral loads in the spleen, lung, and kidney compared to virally infected *Lats1*^*+/+*^ mice. Mechanistically, LATS1 was shown to enhance IFN-I receptor dependent signaling by targeting IFNα/βR2 (IFNAR2) to maximize full activation of signal transducer and activator of transcription 1 (STAT1), a key transcription factor that governs the induction of ISGs (33). Alternatively, our studies found no significant defect in ISG expression upon IFN-I stimulation in *Lats1*^*−/−*^ MEF cells (Fig. S4*C*) to instead suggest that LATS1 operates upstream of IFNAR to control IFN-I activation in cytosolic nucleic acid-sensing PRR pathways as pharmacological inhibition of LATS1 kinase function or genetic ablation of LATS1 resulted in defective activation of the IRF3 transcription factor and subsequent impairment of IFN-I induction upon either cytosolic RNA or DNA pathway stimulation, as well as upon RNA virus or DNA virus infection. Activation of the NF-κB transcription factor and induction of pro-inflammatory cytokines (*e.g.* TNFα, IL-6) upon cytosolic nucleic acid stimulation or viral infections were additionally impaired in LATS1-deficient cells. While our data indicate that LATS1 functions as a positive regulator of IFN-I in cytosolic nucleic acid-sensing PRR signaling pathways, future studies will be needed to fully clarify its role in IFNAR signaling.

**Figure 7:**
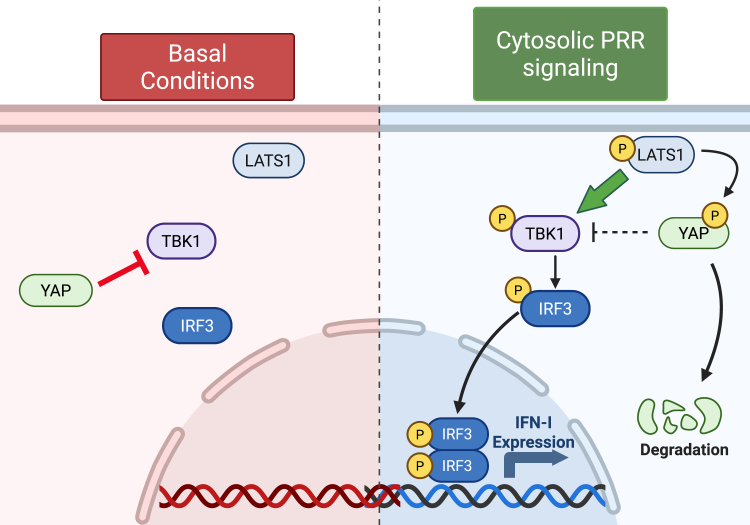
**Model of Hippo kinase LATS1-dependent regulation of IFN-I in cytosolic nucleic acid sensing PRR signaling pathways.** Under basal conditions, YAP operates as a steady-state negative regulator of TBK1-IRF3 signaling in part by associating with TBK1. Upon the detection of RNA or DNA virus genomes by distinct cytosolic PRRs, the Hippo kinase LATS1 undergoes phosphorylation to trigger the phospho-inhibition of YAP, abrogating its ability to antagonize TBK1. Activated LATS1 further associates with TBK1 and licenses TBK1 dependent signaling to IRF3, resulting in the subsequent induction of IFN-I. Figure created with BioRender.

Consistent with a role in cytosolic nucleic acid sensing PRR signaling, our data indicated LATS1 associated with TBK1 upon cytosolic nucleic acid stimulations and that *Lats1*^*−/−*^ MEF cells were defective in triggering the phospho-activation of TBK1, a shared regulatory component in both the RNA and the DNA pathways. While LATS1 did not function as a kinase for TBK1, we instead found that LATS1 kinase activity was important for promoting TBK1 mediated activation of IFN-I. We therefore posit that LATS1 requires its kinase function to phospho-inhibit YAP/TAZ and promote TBK1 signaling to IRF3 resulting in IFN-I induction (Fig. 7). Interestingly, LATS2 was shown to also promote IFN-I activation in the DNA but not the RNA sensing pathway and may possibly be restricted to epithelial keratinocytes (34). In contrast, our data utilizing a dual LATS1/2 kinase inhibitor in *Lats1*^*−/−*^ MEF cells (to presumably target LATS2 function) found that cytosolic RNA pathway-dependent activation of IFN-I was further blunted, suggesting that LATS2 may play a non-redundant role with LATS1 to facilitate IFN-I activation. Future studies in cells doubly deficient in LATS1 and LATS2 will be necessary to understand the exact contributions of LATS2 in innate antiviral signaling pathways.

Beyond its crucial role in antiviral innate immunity, TBK1 is known to influence several cellular processes, including autophagy, inflammation and autoimmunity, metabolic control, and cell survival and cancer pathways (35, 36, 37, 38, 39). As we found LATS1 to promote TBK1 activation and signaling events in the cytosolic nucleic acid sensing pathways, it is tempting to speculate that LATS1 may additionally play a modulatory role in controlling TBK1 function in other physiological or pathological disease states and will be the focus of future investigation.

## Experimental procedures

### Cell culture, reagents, antibodies, and plasmids

*Lats1*^*−/−*^ MEF cells were kindly provided by Dr Xiaolong Yang (Queen’s University). HEK 293T cells, U937 monocytes, and A549 cells were obtained from American Type Culture Collection (ATCC). Cells were cultured in Dulbecco’s Modified Eagle Medium (DMEM) (Corning) (MEF, HEK 293T, A549) or Roswell Park Memorial Institute 1640 (RPMI 1640) (Corning) (U937) supplemented with fetal bovine serum (Gibco) (10%) and penicillin/streptomycin (Gibco) (1%) in 5% CO_2_ at 37 °C.

Poly (I:C) (HMW), LPS-EB, and diABZI were purchased from Invivogen. Oligos for ISD generation were synthesized by Invitrogen. IFN-β enzyme linked immunosorbent assay (ELISA) kit was from ABclonal. Forskolin, Vadimezan (DMXAA), and recombinant murine IFNβ were purchased from MedChemExpress. LATS-IN-1 was obtained from Cayman Chemical Company. Primary antibodies used in this study were FLAG (Sigma Life Sciences); VSV-G, HSV-1 ICP4, HSP90, P65 (Santa Cruz Biotechnology); TBK1, p-TBK1, IRF3, p-IRF3, LATS1, p-LATS1, p-YAP, YAP/TAZ, STING, p-STING, p-P65, β-Actin (Cell Signaling Technology); β-Tubulin, GAPDH, HA (Proteintech); P65 (Santa Cruz Biotechnology). Secondary anti-rabbit, anti-mouse, or anti-rabbit/mouse TrueBlot antibodies conjugated to HRP were purchased from Southern Biotech and Rockland Immunochemicals, respectively.

FLAG-LATS1 was a gift from Dr Xiaolong Yang (Queen’s University). Plasmids encoding TBK1, IRF3, RIG-I, STING, MAVS, IFN-β-luciferase, and Renilla-luciferase were previously described (40, 41). 8xGTIIC-luciferase was a gift from Stefano Piccolo (Addgene plasmid # 34615). FLAG-TBK1-KD (K38A) and FLAG-LATS1 KD (D846A) were generated *via* site-directed mutagenesis (Thermo Scientific).

### Transfections, viral infections and imaging, RNA-mediated interference, CRISPR gene editing

MEF, HEK 293T, and A549 cells were seeded in tissue culture dishes/plates 16 to 18 h prior to polyethylenimine (PEI) (Polysciences, Inc.) or Lipofectamine 2000 (Invitrogen) mediated transfection. U937 cells were seeded in tissue culture dishes and pretreated with phorbol myristate acetate (100 ng/ml, 24 h) (Invivogen) prior to transfection. SeV, VSV-GFP, and HSV-1-GFP infections have been described elsewhere (40, 41). Viral titers were determined using standard plaque assays with Vero cells. MEF cells infected with VSV-GFP or HSV-GFP were subjected to live imaging using the Echo Revolve fluorescent microscope (kindly supported by Dr Shitao Li, Tulane University School of Medicine). For small interfering RNA (siRNA) studies, HEK 293T cells were transfected with 60 pmol of control scrambled siRNA or 3 different siRNA targeting human LATS1 (20 pmol each) (Bioneer) using Lipofectamine 2000 (Invitrogen). To generate LATS1 KO A549 cells, a single guide RNA targeting human LATS1 was cloned into pX330-U6-Chimeric_BB-CBh-hSpCas9 and co-transfected with psPAX2 and pMD2.G packaging and envelope plasmids (Addgene) into HEK 293T cells to produce lentiviral particles to infect A549 cells in the presence of polybrene.

### Immunoblot analysis and immunoprecipitation

For immunoblot analysis, cells were harvested in ice cold NP-40 lysis buffer (50 mM Tris-Cl pH 7.4, 150 mM NaCl, 1 mM EDTA, 1% NP-40) supplemented with complete EDTA-free protease inhibitors (MedChemExpress) as described previously (40, 41). Protein concentrations were determined *via* the BCA protein assay (Thermo Scientific). For immunoprecipitation experiments, precleared lysates from transfected or stimulated cells were incubated overnight with appropriate antibodies at 4 °C followed by the addition of protein A agarose beads (Roche) or protein A/G magnetic beads (MedChemExpress) for 4 h at 4 °C. Captured protein complexes were washed three times with NP-40 lysis buffer containing 250 mM NaCl and then eluted with 2 × Laemmli sample buffer (Bio-Rad) containing β-mercaptoethanol. Samples were boiled at 95 °C for 5 min followed by sodium dodecyl sulfate polyacrylamide gel electrophoresis (SDS-PAGE) and immunoblotting. Proteins were detected *via* enhanced chemiluminescence (Thermo Scientific) using the Amersham Imager 600 (GE Healthcare Life Sciences).

### Luciferase reporter assays

Reporter assays were performed using a dual luciferase assay kit (Promega) 18 to 24 h after transfection with 100 ng firefly luciferase and 10 ng renilla luciferase plasmids co-transfected with the indicated plasmids. Luciferase values were quantified on a luminometer (Berthold) and results for firefly luciferase activity were normalized to renilla luciferase activity.

### RNA isolation and quantitative PCR

RNA was isolated using TRIzol reagent (Invitrogen) and converted to cDNA using ABScript III RT mix (ABclonal). Quantitative PCR (q-PCR) was performed using SYBR green (ABclonal) in a CFX96 thermocycler (Bio-Rad). Transcript abundance was first normalized to that of mRNA encoding the ribosomal protein L32 for murine mRNA transcripts or 36B4 for human mRNA transcripts, then normalized against values for unstimulated controls calculated *via* the 2^-ΔΔCt^ method. Murine primer sequences were as follows:

Ifnb Fwd: AGCTCCAAGAAAGGACGAACAT, Rev: GCCCTGTAGGTGAGGTTGATCT; Cxcl10 Fwd: CCAGT GAGAATGAGGGCCATA, Rev: TCGTGGCAATGA TCTCAACAC; Ccl5 Fwd: GCCCACGTCAAGGAGTATTTCTA, Rev: ACACACTTGGCGGTTCCTTC; RPL32 Fwd: AAGCGAAACTGGCGGAAAC, Rev TAACCGATGTTGG GCATCAG; Isg15 Fwd: CAGGACGGTCTTACCCTTTCC, Rev: AGGCTCGCTGCAGTTCTGTAC; Il6 Fwd: TCTGCAAGAGACTTCCATCCAGTTGC, Rev: AGCCTCCGACTTGTGAAGTGGT; Tnfa Fwd: GGTGATCGGTCCCCAAAGGGATGA, Rev TGGTTTGCTACGACGTGGGCT; Cyr61 Fwd: GGATCTGTGAAGTGCGTCCT, Rev: CTGCATTTCTTGCCCTTTTT.

Ctgf Fwd: TGACCTGGAGGAAAACATTAAGA, Rev: AGCCCTGTATGTCTTCACACTG.

Human primer sequences were as follows:

IFNB1 Fwd: TGTGGCAATTGAATGGGAGGAGGCTTGA, Rev: CGGCGTCCTCCTTCTGGAACTG; RPLP0/36B4 Fwd: TCGAACACCTGCTGGATGAC, Rev: CCACGCTGCTGAACATGC; CCL5 Fwd: CGCTGTCATCCTCATTGCTA, Rev: GGGTGACAAAGACGACTGCT; IL-6 Fwd: AAAGAGGCACTGGCAGAAAA, Rev: TTTCACCAGGCAAGTCTCCT: ISG54 Fwd: TGCAACCTACTGGCCTATCTA, Rev: CAGGTGACCAGACTTCTGATT; CXCL10 Fwd: ATGAATCAAACTGCGATTCCTGATTTGCTGC, Rev: TTAAGGAGATCTTTTAGCCATTTCCTTGC.

### Statistical analysis

Quantitative data are expressed as mean -fold increase ± S.E. relative to control levels from a representative experiment performed 2 to 3 times. Statistical significance was determined using student’s *t* test (∗∗∗*p* < 0.001, ∗∗*p* < 0.01, and ∗*p* < 0.05)

## Data availability

All data described are contained within the manuscript.

## Supporting information

This article contains supporting information.

## Conflict of interest

The authors declare that they do not have any conflicts of interest with the content of this article.
