# Supplementary material for: Cytosolic nucleic acid sensing triggers type I interferon activation via the Hippo kinase LATS1
Source: J Biol Chem. 2026 Jan 23;302(3):111204. doi: 10.1016/j.jbc.2026.111204 (PMC12927312; doi:10.1016/j.jbc.2026.111204)
Supplement: Supporting information [file mmc1.pdf]

Supporting Information 1: Virus infection and cytosolic nucleic acid sensing triggers hippo pathway activation in a LATS1 dependent manner.

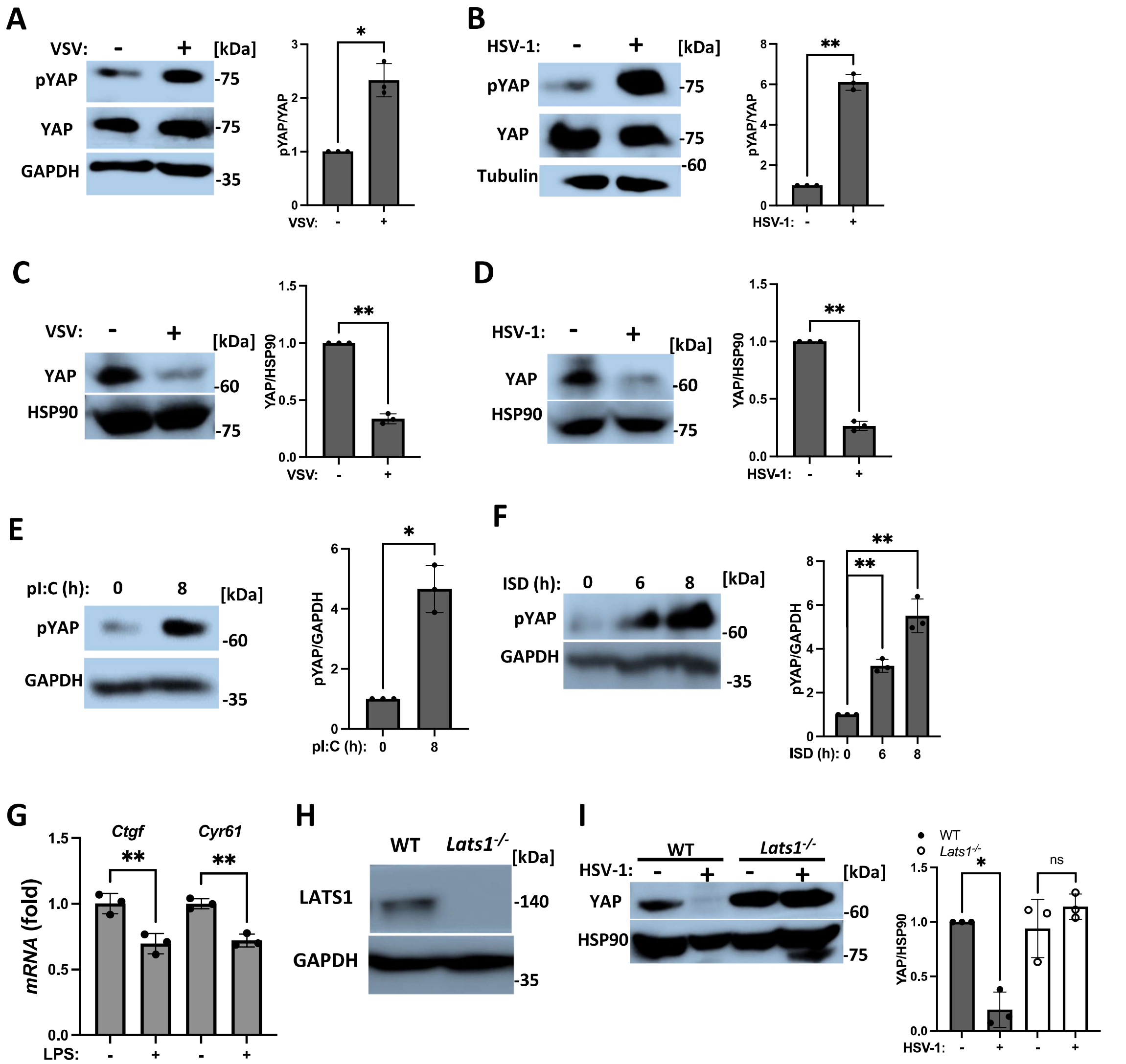

Supporting Information 1: Virus infection and cytosolic nucleic acid sensing triggers Hippo pathway activation in a LATS1 dependent manner.

(A-B) Immunoblot (left panels) and relative quantification (right panels) of YAP (S127) phosphorylation in MEF cells infected with VSV (MOI 0.1) (A) or HSV-1 (MOI 0.2) (B) for 18 h and treated with MG132 (10 µM) proteasomal inhibitor. (C-D) Immunoblot (left panels) and relative quantification (right panels) of YAP expression in MEF cells infected with VSV (MOI 0.5) (C) or HSV-1 (MOI 1.0) (D) for 24 h. (E-F) Immunoblot (left panels) and relative quantification (right panels) of YAP (S127) phosphorylation in MEF cells transfected with pI:C (E) or ISD (F) (2 µg/mL each) for the indicated times. (G) qPCR of TEAD regulated genes Ctgf and Cyr61 in MEF cells stimulated with lipopolysaccharide (LPS) (100 ng/mL; 5 h). (H) Immunoblot of LATS1 expression in Wild type (WT) and *Lats1*$^{-/-}$ MEF cells. (I) Immunoblot (left panel) and relative quantification (right panel) of YAP expression in WT and *Lats1*$^{-/-}$ MEF cells infected with HSV-1 (MOI 1.0; 24 h). Statistical significance was determined using student's t-test (***P<0.001, **P<0.01, and *P<0.05).

Supporting Information 2: Pharmacological inhibition of LATS1 impedes cytosolic nucleic acid sensing PRR activation of IFN-I.

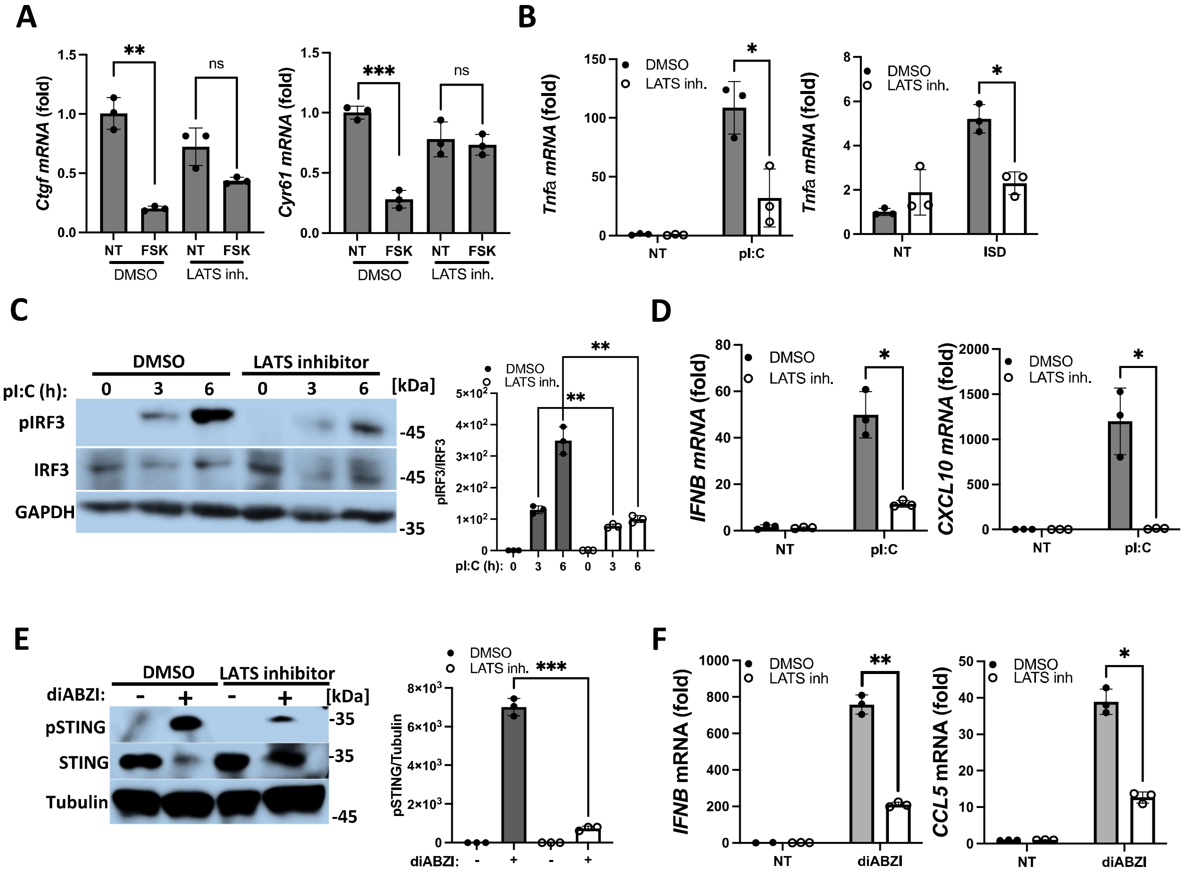

Supporting Information 2: Pharmacological Inhibition of LATS impedes cytosolic nucleic acid sensing PRR activation of IFN-I.

(A) qPCR analysis of Ctgf and Cyr61 expression in MEF cells pretreated with DMSO or LATS inhibitor (20 µM; 2.5 h) followed by stimulation with the Hippo pathway activator, Forskolin (FSK) (10 µM; 2 h). (B) qPCR of Tnfa mRNA in MEF cells pretreated with DMSO or LATS1 inhibitor as in A, followed by pI:C transfection (2 µg/mL; 3.5 h) (left panel) or ISD transfection (2 µg/mL; 4 h) (right panel). (C) Immunoblot analysis (left panel) and relative quantification (right panel) of IRF3 (S396) phosphorylation in U937 cells pretreated with DMSO or LATS inhibitor (10 µM; 2.5 h) followed by pI:C transfection (2 µg/mL) for the indicated times. (D) qPCR of IFNB mRNA (left panel) or CXCL10 mRNA (right panel) in U937 cells pretreated with DMSO or LATS1 inhibitor as in C, followed by pI:C transfection (2 µg/mL; 16 h). (E) Immunoblot analysis (left panel) and relative quantification (right panel) of STING (S366) phosphorylation in U937 cells pretreated with DMSO or LATS1 inhibitor as in C, followed by stimulation with the STING agonist diABZI (1 µM; 16 h). (F) qPCR analysis of IFNB mRNA (left panel) or CCL5 mRNA (right panel) in U937 cells pretreated with DMSO or LATS1 inhibitor as in C, followed by diABZI stimulation (100 nM; 8 h). Statistical significance was determined using student's t-test (***P<0.001, **P<0.01, and *P<0.05).

Supporting Information 3: LATS1 is required for IFN-I activation.

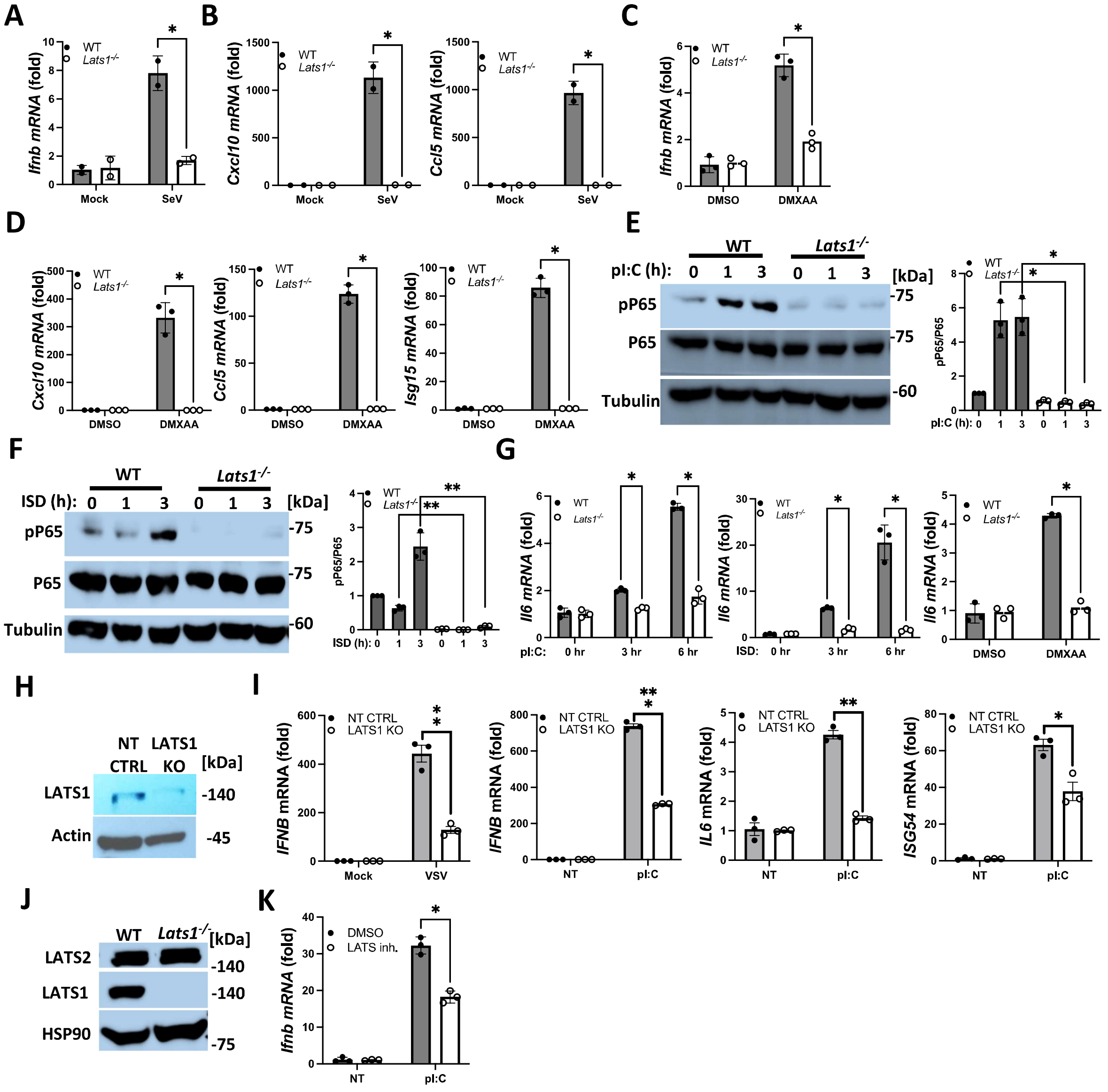

Supporting Information 3: LATS1 is required for IFN-I activation.

(A) qPCR analysis of Ifnb expression in WT and *Lats1*$^{-/-}$ MEF cells infected with Sendai virus (SeV) (MOI 0.1; 24 h). (B) qPCR analysis of Cxcl0 mRNA (left panel) or Ccl5 mRNA (right panel) expression in WT and *Lats1*$^{-/-}$ MEF cells infected with SeV as in A. (C) qPCR of Ifnb mRNA in WT and *Lats1*$^{-/-}$ MEF cells pretreated with DMSO vehicle control or the STING ligand, DMXAA (100 µg/mL; 4 h). (D) qPCR of Cxcl10 mRNA (left panel), Ccl5 mRNA (central panel), and Isg15 mRNA (right panel) in WT and *Lats1*$^{-/-}$ MEF cells treated with DMXAA as in S3C. (E-F) Immunoblot analysis (left panels) and relative quantification (right panels) of P65 (S536) phosphorylation in WT and *Lats1*$^{-/-}$ MEF cells transfected with pI:C (2 µg/mL) (E) or ISD (4 µg/mL) (F) for the indicated times. (G) qPCR analysis of Il6 mRNA expression in WT and *Lats1*$^{-/-}$ MEF cells transfected with pI:C, ISD, or treated with DMXAA as in 3G-H, or S3C. (H) Immunoblot of LATS1 expression in control or LATS1 KO A549 cells. (I) qPCR analysis of IFNB mRNA in control or LATS1 KO A549 cells infected with VSV (MOI 0.1; 16 h) or transfected with pI:C (500 ng/mL; 3 h) (left panels) and IL6 and ISG54 mRNA expression in WT and LATS1 KO A549 cells transfected with pI:C (500 ng/mL; 3h) (right panels). (J) Immunoblot analysis of LATS1 and LATS2 in WT and *Lats1*$^{-/-}$ MEF cells. (K) qPCR analysis of Ifnb expression in *Lats1*$^{-/-}$ MEF cells pretreated with DMSO or LATS inhibitor (10 µM; 2.5 h) followed by pI:C transfection (8 µg/mL; 6 h). Statistical significance was determined using student's t-test (***$P<0.001$, **$P<0.01$, and *$P<0.05$).

Supporting Information 4: LATS1 facilitates TBK1 activation and signaling events.

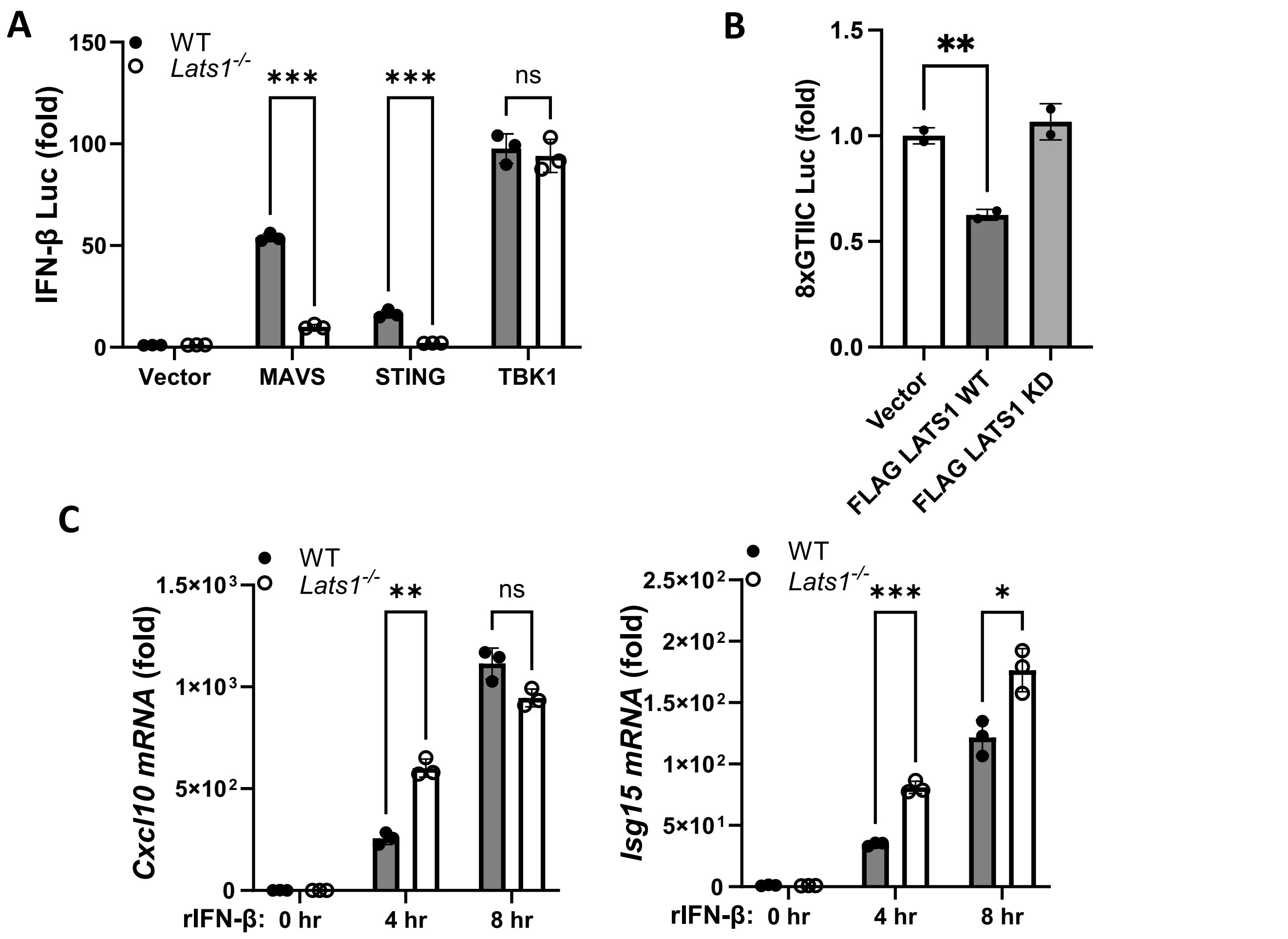

Supporting Information 4: LATS1 facilitates TBK1 activation and signaling events.
(A) IFN-β luciferase reporter assay in WT and *Lats1*$^{-/-}$ MEF cells transfected with plasmids encoding MAVS, STING, and TBK1. (B) Luciferase reporter assay measuring YAP/TAZ transcriptional activity in HEK 293T cells transfected with plasmids encoding WT LATS1 or kinase dead (KD) LATS1 (D846A). (C) qPCR analysis of Cxcl0 mRNA (left panel) or Isg15 mRNA (right panel) expression in WT and *Lats1*$^{-/-}$ MEF cells stimulated with recombinant IFN-β (100 U/mL) for the indicated times. Statistical significance was determined using student's t-test (***P<0.001, **P<0.01, and *P<0.05).
